# Using Bayesian adaptive designs to improve phase III trials: a respiratory care example

**DOI:** 10.1186/s12874-019-0739-3

**Published:** 2019-05-14

**Authors:** Elizabeth G. Ryan, Julie Bruce, Andrew J. Metcalfe, Nigel Stallard, Sarah E. Lamb, Kert Viele, Duncan Young, Simon Gates

**Affiliations:** 1 Warwick Clinical Trials Unit, Warwick Medical School, University of Warwick, Coventry, CV4 7AL UK; 2 Cancer Research UK Clinical Trials Unit, Institute of Cancer and Genomic Sciences, University of Birmingham, Birmingham, UK; 3 Department of Trauma and Orthopaedic Surgery, University Hospital Coventry & Warwick, Coventry, UK; 4 Statistics and Epidemiology, Division of Health Sciences, Warwick Medical School, University of Warwick, Coventry, UK; 5 Centre for Rehabilitation Research and Centre for Statistics in Medicine, Nuffield Department of Orthopaedics Rheumatology & Musculoskeletal Sciences, Botnar Research Centre, University of Oxford, Oxford, UK; 6 Berry Consultants, Austin, TX USA; 7 Nuffield Department of Clinical Neurosciences, University of Oxford, John Radcliffe Hospital, Oxford, UK

**Keywords:** Bayesian sequential design, Interim analyses, Randomised controlled trials, Critical care

## Abstract

**Background:**

Bayesian adaptive designs can improve the efficiency of trials, and lead to trials that can produce high quality evidence more quickly, with fewer patients and lower costs than traditional methods. The aim of this work was to determine how Bayesian adaptive designs can be constructed for phase III clinical trials in critical care, and to assess the influence that Bayesian designs would have on trial efficiency and study results.

**Methods:**

We re-designed the High Frequency OSCillation in Acute Respiratory distress syndrome (OSCAR) trial using Bayesian adaptive design methods, to allow for the possibility of early stopping for success or futility. We constructed several alternative designs and studied their operating characteristics via simulation. We then performed virtual re-executions by applying the Bayesian adaptive designs using the OSCAR data to demonstrate the practical applicability of the designs.

**Results:**

We constructed five alternative Bayesian adaptive designs and identified a preferred design based on the simulated operating characteristics, which had similar power to the original design but recruited fewer patients on average. The virtual re-executions showed the Bayesian sequential approach and original OSCAR trial yielded similar trial conclusions. However, using a Bayesian sequential design could have led to a reduced sample size and earlier completion of the trial.

**Conclusions:**

Using the OSCAR trial as an example, this case study found that Bayesian adaptive designs can be constructed for phase III critical care trials. If the OSCAR trial had been run using one of the proposed Bayesian adaptive designs, it would have terminated at a smaller sample size with fewer deaths in the trial, whilst reaching the same conclusions. We recommend the wider use of Bayesian adaptive approaches in phase III clinical trials.

**Trial registration:**

OSCAR Trial registration ISRCTN, ISRCTN10416500. Retrospectively registered 13 June 2007.

**Electronic supplementary material:**

The online version of this article (10.1186/s12874-019-0739-3) contains supplementary material, which is available to authorized users.

## Background

Phase III randomised controlled trials (RCTs) are typically long and expensive, restricting their use and resulting in long lead times to answer important clinical questions [1]. Traditional phase III design methods require specification of the sample size in advance. This can be inefficient when limited information is available at the design stage, especially regarding the likely effect size. Researchers and funders have recognised the need to use more efficient trial designs, yet the majority of trials continue to use traditional methods. Adaptive and sequential trial designs have been described and even recommended by bodies such as the US Food and Drug Administration [2], but their use remains sporadic amid uncertainty about their utility outside of early phase trials.

Sequential adaptive designs allow repeated interim analyses during the trial to decide whether it should continue or terminate due to sufficient evidence to reach a conclusion. The timing and criteria for these decisions must be specified before the trial begins. Sequential designs can offer a more efficient approach for conducting RCTs and frequently result, on average, in smaller and shorter trials than traditional approaches. Sequential designs may be implemented using frequentist methods, which typically use null hypothesis testing, or Bayesian methods.

The use of Bayesian statistical methods for designing and analysing RCTs has increased (e.g., [3–6]). We concentrate here on Bayesian methods, because they have a number of advantages, particularly for adaptive trials. Bayesian statistics provide a formal method for updating information about the treatment effect as new data are observed, and hence are well suited to interim analyses with accumulating information. The results of Bayesian analyses may also be easier to interpret than frequentist analyses as they can provide the probability of various estimates of the unknown treatment effect. The posterior distribution can also provide probabilistic statements about other measures of interest, such as adverse event rates and the dose-response relationship.

Bayesian approaches require specification of a prior distribution for the possible values of the unknown treatment effect, thereby accounting for uncertainty in its value. The prior distribution can incorporate previous information. Accumulating trial information is combined with this prior to produce a posterior distribution that summarises the current state of knowledge about the treatment effect. This updating occurs at each interim analysis.

The posterior distribution drives key decisions at each interim analysis, such as stopping for trial success. Predictive probabilities can also be obtained from the posterior, such as the probability that the trial will be successful if it continues to completion. These measures, which are more clinically relevant than *p*-values, can be used to decide whether the trial should stop at an interim analysis in what will be termed a “Bayesian sequential design” [7].

Bayesian adaptive trial designs are increasingly being used in early phase trials, but their use in phase III trials is more limited. The few published works mostly consist of trial protocols [8] or are re-executions of completed traditionally-designed trials using Bayesian adaptive designs for comparative purposes (e.g., [9–12]). Few published phase III trials have used Bayesian adaptive methods from the design phase (e.g., [3, 5, 6]).

The aim of this work was to explore the implementation of Bayesian sequential designs for phase III trials in critical care. Using an example from a recent critical care trial, we demonstrate how a Bayesian sequential design can be constructed, and illustrate the choices required during the design phase. The operating characteristics of the designs were studied via simulation and virtual re-executions of the trial were performed using the Bayesian designs and actual trial data. These were conducted to establish the efficiency of such designs, and demonstrate how these designs can be implemented and the decisions that would be made in a real-world trial during the interim analyses.

## Materials and methods

### Case study

The High Frequency OSCillation in Acute Respiratory distress syndrome (ARDS) study (OSCAR) [13] compared conventional positive pressure ventilation (control) with high frequency oscillatory ventilation (HFOV) in adults with ARDS. The primary outcome was mortality at 30 days. The planned sample size was 1006 patients (503 in each arm). This gave 80% power to detect a 9% reduction from a control group 30-day mortality of 45% with a significance level of 5%, assuming 3% dropout. Recruitment occurred from 7 December 2007 to 31 July 2012.

Two formal interim analyses were planned in the OSCAR trial at approximately one third and two thirds of the way through recruitment. The design allowed for an additional interim analysis halfway through recruitment if the Data Monitoring and Ethics Committee (DMEC) requested closer monitoring. No formal stopping rules were used, but O’Brien-Fleming alpha-spending functions were used to provide guideline critical values for early success stopping and control type I error. These values were calculated for designs where two or three interim analyses may be performed. The trial could be stopped or modified by the DMEC if the treatments were convincingly different in terms of 30-day mortality, or for safety reasons. The DMEC requested sample size re-estimation to be performed at a planned interim analysis, due to slow recruitment. The trial statistician became unblind to the control arm primary outcome data and calculated a revised sample size of 401 patients per arm. This calculation assumed a 10% absolute change in mortality, with 80% power, a 5% significance level, and 3% dropout.

OSCAR randomised 795 patients from 29 hospitals and did not find any evidence that HFOV was superior in the primary outcome: 166/398 (41.7%) HFOV patients and 163/397 (41.1%) control patients died within 30-days (*p* = 0.85).

### Potential adaptations and candidate designs

Alternative Bayesian designs for the OSCAR trial were constructed by identifying adaptations, independently of the trial data, to improve the trial’s efficiency. The OSCAR trial allowed for early success stopping but did not allow early stopping for statistical futility. Early stopping for statistical futility can be useful in some trials as it can preserve resources that could instead be used on more promising treatments and prevent patients from being given an ineffective experimental treatment. The Bayesian sequential designs allowed early stopping for lack of benefit or evidence of success. The number and timing of the interim analyses were investigated. Interim analyses could occur based on either calendar time or number of patients recruited.

The designs were constructed by a statistician (EGR) who was blind to the trial results and relied on the OSCAR protocol and statistical analysis plan. Feedback was provided from two clinical academics with experience in running RCTs (JB and AJM) who were also independent from the original trial and blinded to the results.

The maximum sample size was specified to be the same as the original planned sample size (*N* = 1006). The clinical academics provided key information on the minimal sample size for interpretability and on logistics. They indicated that success stopping should not occur before enrolling half of the original proposed sample size and that more than three interim analyses would be too burdensome. This led to a final candidate set of six designs (see Table 1) including a non-adaptive/fixed design with a Bayesian final analysis (Design 1) for comparative purposes. The stopping boundaries in Table 1 are described in the “Decision criteria” section.

**Table 1:** Candidate Bayesian sequential designs generated for OSCAR

Design	Interim	Timing of interim (information fraction)^a^	Can stop for success	Can stop for futility	Success stopping boundaries^b^	Futility stopping boundaries^c^
1	NA (fixed design)	NA	NA	NA	NA	NA
2	1	250 (1/4)	No	Yes	NA	F_1_ = 0.05
	2	500 (1/2)	Yes	Yes	S_2_ = 0.99	F_2_ = 0.1
	3	750 (3/4)	Yes	Yes	S_3_ = 0.98	F_3_ = 0.15
3	1	335 (1/3)	No	Yes	NA	F_1_ = 0.05
	2	670 (2/3)	Yes	Yes	S_2_ = 0.99	F_2_ = 0.1
4	1	335 (1/3)	No	Yes	NA	F_1_ = 0.05
	2	500 (1/2)	Yes	Yes	S_2_ = 0.99	F_2_ = 0.1
	3	670 (2/3)	Yes	Yes	S_3_ = 0.98	F_3_ = 0.15
5	1	503 (1/2)	Yes	Yes	S_1_ = 0.99	F_1_ = 0.05
	2	755 (3/4)	Yes	Yes	S_2_ = 0.98	F_2_ = 0.1
6	1	503 (1/2)	Yes	Yes	S_1_ = 0.99	F_1_ = 0.05
	2	755 (3/4)	Yes	Yes	S_2_ = 0.98	F_2_ = 0.1
	3	880 (7/8)	Yes	Yes	S_3_ = 0.98	F_3_ = 0.15

^a^The timing of the interims was based on the number of patients recruited

^b^S_i_ is the stopping boundary for success at the *i*-th interim analysis. ^c^ F_i_ is the stopping boundary for futility at the *i*-th interim analysis. The stopping boundaries are described in the “Decision criteria” section

### Software and simulation settings

Simulations of the trial designs were performed using the Fixed and Adaptive Clinical Trial Simulator (FACTS) program version 6.1 [14]. For each design 10,000 example trials were simulated assuming a true effect size. These simulations allowed the distribution of final sample size and duration to be estimated, and quantified type I error and power. The simulations also provided insights into how the operating characteristics were affected if the trial conditions were not as expected, e.g., slower recruitment, or unexpected harm.

The Bayesian sequential designs were constructed as one-sided superiority studies as we were interested in showing reduction in mortality in HFOV over the control. A range of plausible scenarios were simulated for each design to investigate the operating characteristics of the designs under a range of true effect sizes (see Table 2). There was some uncertainty regarding the control primary outcome rate, so this rate was varied in the plausible scenarios.

**Table 2:** Operating characteristics for the proposed Bayesian sequential designs for the OSCAR trial^a^

Design	Scenario: control vs HFOV primary outcome rate	Average duration (weeks)	Average sample size (SD)	Proportion stopped early for success	Overall Proportion Successful ^b^	Proportion stopped early for futility
Design 1: Fixed design	No difference: 45% vs 45%	196	1006 (0)	NA	*0.0283*	NA
	Target difference: 45% vs 36%	196	1006 (0)	NA	***0.8219***	NA
	Small difference: 45% vs 40%	196	1006 (0)	NA	0.3503	NA
	Large difference: 45% vs 30%	196	1006 (0)	NA	0.9979	NA
	Treatment harmful: 45% vs 50%	196	1006 (0)	NA	0.0003	NA
Design 2: Interim analysis at 250, 500 and 750 patients	No difference: 45% vs 45%	103	519 (236)	0.0123	*0.0268*	0.8956
	Target difference: 45% vs 36%	145	730 (227)	0.5319	***0.7793***	0.1434
	Small difference: 45% vs 40%	141	719 (260)	0.1607	0.3302	0.4828
	Large difference: 45% vs 30%	114	560 (127)	0.9592	0.9932	0.0058
	Treatment harmful: 45% vs 50%	75	367 (155)	0.0004	0.0004	0.9949
Design 3: Interim analysis at 335 and 670 patients	No difference: 45% vs 45%	130	664 (207)	0.0070	*0.0264*	0.8123
	Target difference: 45% vs 36%	163	828 (179)	0.4314	***0.8079***	0.0829
	Small difference: 45% vs 40%	161	825 (207)	0.1151	0.3431	0.3593
	Large difference: 45% vs 30%	139	696 (90)	0.9214	0.9971	0.0019
	Treatment harmful: 45% vs 50%	109	555 (170)	0.0000	0.0005	0.9841
Design 4: Interim analysis at 335, 500 and 670 patients	No difference: 45% vs 45%	112	564 (202)	0.0087	*0.0229*	0.8578
	Target difference: 45% vs 36%	147	741 (230)	0.4723	***0.7873***	0.1227
	Small difference: 45% vs 40%	146	742 (242)	0.1362	0.3334	0.4371
	Large difference: 45% vs 30%	114	557 (131)	0.9334	0.9958	0.0033
	Treatment harmful: 45% vs 50%	91	454 (108)	0.0002	0.0003	0.9895
Design 5: Interim analysis at 503 and 755 patients	No difference: 45% vs 45%	138	712 (158)	0.0099	*0.0249*	0.8637
	Target difference: 45% vs 36%	160	812 (176)	0.5237	***0.813***	0.0922
	Small difference: 45% vs 40%	163	834 (174)	0.1501	0.3426	0.4062
	Large difference: 45% vs 30%	134	667 (139)	0.9596	0.9968	0.0015
	Treatment harmful: 45% vs 50%	126	645 (129)	0.0002	0.0005	0.9915
Design 6: Interim analysis at 503, 755 and 880 patients	No difference: 45% vs 45%	136	702 (143)	0.0130	*0.0270*	0.9381
	Target difference: 45% vs 36%	158	792 (159)	0.6320	***0.7990***	0.1415
	Small difference: 45% vs 40%	158	810 (156)	0.2035	0.3376	0.5420
	Large difference: 45% vs 30%	134	664 (132)	0.9847	0.9966	0.0027
	Treatment harmful: 45% vs 50%	127	644 (127)	0.0002	0.0005	0.9972

^a^The “proportions” in columns 5–7 refer to the proportion of the 10, 000 simulated trials for each scenario, and the averages and standard deviations (SD) are over the 10, 000 simulated trials. ^b^The one-sided simulated type I error is italicised; the power is boldfaced and italicised

The recruitment rate was simulated in FACTS using a mean of 5.5 participants/week, based on the original projected recruitment rates for OSCAR. The same assumed dropout rate was used as for OSCAR. The operating characteristics were also studied assuming slower recruitment (2/week), faster recruitment (11/week), and no dropouts.

Non-informative prior distributions were used for the primary outcome rate for each arm, corresponding to all response rates between 0 and 100% being equally likely. More informative (and more realistic) priors based on previous studies were also investigated (see Additional file 1).

### Decision criteria

“Trial success” was defined as declaring superiority of HFOV. The stopping criteria for statistical futility were based on the posterior predictive probability of trial success at the maximum sample size, which is denoted by P_max_. P_max_ incorporates accumulated complete data, uncertainty in patients enrolled without complete follow-up, and uncertainty in future patients up to the maximum sample size [9]. The trial was stopped early for futility if P_max_ was less than a futility threshold *F*_*i*_ at interim analysis *i*. For the purposes of simulating trial duration, if a trial stopped for futility the trial was assumed to cease immediately. If the intervention was causing harm, then the trial would be stopped for “futility” at the interim analyses.

The stopping criteria for success were based on the posterior predictive probability of trial success at the current sample size, after accounting for uncertainty in enrolled patients without complete follow-up. If this probability, P_curr_, was greater than the success threshold *S*_*i*_ at interim analysis *i*, accrual stopped for success. All incomplete patients were followed up, after which the final analysis was conducted. Operating characteristics were calculated assuming the futility and success stopping rules would always be followed. The original OSCAR trial did not have binding stopping rules.

Using values based on previous studies [9, 10, 15] a range of potential success and futility threshold values were explored. For the candidate designs in Table 1, threshold values were chosen that produced similar power to the original design and a one-sided type I error of approximately 2.5%. Table 1 gives the stopping boundaries for each design.

The trial was deemed successful at the final analysis if the posterior probability that HFOV had a lower 30-day mortality rate was above 0.975. This value was chosen based on the clinical academics’ preferences and by considering the power and simulated type I error it produced. The same value was used for each Bayesian design.

The type I error was calculated from the simulations under the null hypothesis scenario of no difference, estimating the type I error rate as the proportion of such simulations that falsely declared HFOV superior. The power was calculated as the proportion of simulations that concluded that HFOV was superior under the target difference of 9%.

### Identification of preferred design

The designs’ operating characteristics were presented to the clinical academics to identify a preferred design that they felt could have been implemented. It was desirable to have a design which offered high power, low type I error, and minimised the sample size. These present a trade-off as designs which aggressively minimise sample size may result in lower power.

### Virtual re-execution of designs

A virtual re-execution of the OSCAR trial was performed to illustrate the application of the Bayesian sequential designs to a real-world trial. The trial data were read into FACTS and each of the Bayesian sequential designs were implemented. The trial data were read in using the original sequence of patients in recruitment order with the interim analyses being performed after the appropriate number of recruits for each design. At each interim analysis, accumulated data were analysed to determine whether the trial should be terminated early. It was assumed that there were no delays in having the endpoint data available for analysis. These re-executions represent the analysis of a single realisation of the trial. If a different trial dataset had been used, different conclusions would have been drawn from using these particular Bayesian designs. The OSCAR trial data represent the only existing dataset of patients that were actually recruited to this trial, in contrast to simulated datasets, which may fail to capture some important aspect. The virtual re-executions therefore show what would have happened if a different trial design had been used when running the OSCAR trial.

## Results

### Design simulations and operating characteristics

Table 2 presents the average sample size, average duration, type I error, and power for each design and scenario. Distributions for the sample size and study duration over the 10,000 simulations are shown in Additional file 2: Figure S1 and Figure S2. Table 2 also presents the proportion of simulations stopped early for success or futility. There was little variation in the operating characteristics when the prior or accrual assumptions were varied (results not presented).

The Bayesian sequential designs had around 80% power and acceptable simulated type I errors of 2.3—2.7%, while saving on the sample size. Under the null scenario, the average sample sizes were reduced by approximately 300–500 patients. Under the target difference of a 9% reduction in 30-day mortality, the average sample sizes were reduced by approximately 200–300 patients. Similar sample size reductions were observed when a small positive effect was assumed. The average sample size was reduced by 300–450 patients when a large positive effect was assumed.

The designs performed well in terms of safety and efficiency, in that they stopped earliest for a harmful effect, followed by either a large positive effect or no effect. Uncertainty in the control arm rate had little impact (see in Additional file 3: Table S1).

Design 5, which had interim analyses at 503 and 755 patients recruited, was chosen independently by JB and AJM, since it had the highest power of the Bayesian designs at 81.3% and a low type I error rate of 2.49%. The clinical academics also preferred the timing of these interim analyses.

### Re-execution of the OSCAR trial

#### Interim analyses

The virtual executions of Designs 2–5 are presented in Table 3. Design 1 (fixed design) is not presented. Design 6 was not executed as the trial would have stopped before interim analysis 3, giving the same results as Design 5. Sensitivity analyses were performed using alternatives to the default non-informative priors, but little differences were seen between the analyses (results not presented).

**Table 3:** Virtual executions of the OSCAR trial using the Bayesian sequential designs

		Design 2: Interim analysis at 250, 500 and 750 patients	Design 3: Interim analysis at 335 and 670 patients	Design 4: Interim analysis at 335, 500 and 670 patients	Design 5: Interim analysis at 503 and 755 patients
Interim 1	Decision	Stopping criteria not met	Stopping criteria not met	Stopping criteria not met	Stopping criteria not met
	Randomisation allocation (control: HFOV)	129: 121	174: 161	174:161	251:252
	Primary outcome (control; HFOV)	49/118 (41.5%); 44/113 (38.9%)	70/165 (42.4%); 57/153 (37.3%)	70/165 (42.4%); 57/153 (37.3%)	98/233 (42.1%); 93/240 (38.8%)
	Posterior probability HFOV superior	0.6406	0.8222	0.8222	0.7556
	P_max_^a^	0.2410	0.3958	0.3958	0.1747
Interim 2	Decision	Stopping criteria not met	Stop for futility	Stopping criteria not met	Stop for futility
	Randomisation allocation (control: HFOV)	249:251	339:331	249: 251	380:375
	Primary outcome (control; HFOV)	96/230 (41.7%); 93/239 (38.9%)	136/330 (41.2%); 129/322 (40.1%)	96/230 (41.7%); 93/239 (38.9%)	154/375 (41.1%); 152/364 (41.8%)
	Posterior probability HFOV superior	0.7350	0.6490	0.7350	0.4146
	P_max_^a^	0.1315	0.0128	0.1315	0.0000
Interim 3	Decision	Stop for futility	NA	Stop for futility	NA
	Randomisation allocation (control: HFOV)	377:373	NA	339:331	NA
	Primary outcome (control; HFOV)	154/372 (41.4%); 152/363 (41.9%)	NA	136/330 (41.2%); 129/322 (40.1%)	NA
	Posterior probability HFOV superior	0.4372	NA	0.6490	NA
	P_max_^a^	0.0000	NA	0.0128	NA

^a^Posterior predictive probability of having a successful trial if continue to maximum recruitment

In the interim analyses, the posterior probability that HFOV was superior ranged from 0.44–0.82 across the different interim analysis points. As the trial progressed, there was a decrease in the posterior predictive probabilities of having a successful trial if the trial continued to completion with 1006 patients, and all designs stopped early for futility. The stopping boundary for futility at the second interim analysis was 0.1 for all designs, and was met by Designs 3, 5 and 6. Designs 2 and 4, with the second interim analysis taken earlier, did not meet the stopping boundaries for futility until their third interim analysis. P_max_ (the predictive probability of success if the trial continued to its maximum sample size) did not drop below 0.1 until 546 patients had been recruited (*P*_max_ = 0.0893 at *N* = 546).

#### Final analyses

The analyses based on the final data, including follow-up, from each Bayesian sequential design are presented in Table 4 along with the original trial results and the savings in recruitment for these trial data. Results for Designs 4 and 6 are not presented as these are the same as for Designs 3 and 5, respectively. There was little variation in the results across the designs – the relative risks (RRs) ranged from 0.99–1.02, and the posterior probabilities that HFOV was superior ranged from 0.40–0.53.

**Table 4:** Final analyses based on the resulting sample collected from each design

	OSCAR trial	Design 2	Design 3	Design 5
Primary outcome (control; HFOV)	163/397 (41.1%); 166/398 (41.7%)	154/377 (40.8%); 156/373 (41.8%)	138/339 (40.7%); 134/331 (40.5%)	156/380 (41.1%); 157/375 (41.9%)
RR (95% CI)	1.02 (0.86, 1.20)	1.02 (0.86, 1.22)	0.99 (0.83, 1.20)	1.02 (0.86, 1.21)
Posterior probability HFOV superior	0.46	0.40	0.53	0.40
Number of deaths in trial	329	310	272	313
Number randomised^a^	795	750	670	775
Recruitment savings from original sample size of N = 1006	211	256	336	231
Recruitment savings from achieved OSCAR sample size of *N* = 795	NA	45	125	20
Accrual Duration (weeks)^b^	243	227	203	228

^a^Based on the number of patients required to trigger the interim analyses at which the trial was stopped. ^b^These numbers are based on the randomisation date for the patient that triggered the interim analysis at which the trial was stopped

The results given in Table 4 show that the re-executions using the different designs based on this single set of trial data reflect the operating characteristics presented and discussed above. With the data observed, each of the proposed Bayesian sequential designs would have saved on overall trial duration, sample size and number of deaths relative to the actual trial. The Bayesian sequential designs could have shortened the trial duration by between 15 and 40 weeks and recruited 231–336 fewer patients than the target sample size of *N* = 1006 and 20–125 fewer patients than the 795 that the OSCAR trial achieved.

Designs 3 and 4 performed best in terms of reducing the number of patients randomised and the trial duration by stopping the trial earliest for futility. These are the Bayesian versions of the OSCAR design that was originally proposed. In the re-executions, all of the Bayesian designs would have terminated the trial with fewer patient deaths than the trial that was actually conducted.

Care should be taken not to over-interpret these results since they represent the analysis of a single dataset. However, the data are the only information on patients recruited to this trial that actually exists, and re-executing the trial with alternative designs tells us how these would have performed in reality. To fully understand the real-life efficiency savings that these designs may lead to, they would have to be run on a large number of trials. It should be recognised that a large number of phase III trials fail to detect differences between treatment arms and therefore the example chosen is a common scenario, and more widespread use of futility stopping would likely result in efficiency gains across a broad portfolio of trials.

## Discussion

### Summary

We have demonstrated how Bayesian sequential designs, a type of adaptive design, could be implemented for a phase III trial in acute respiratory distress syndrome (ARDS). We outlined the process involved in constructing the sequential designs, and demonstrated their operating characteristics under a range of scenarios. These showed potential advantages over the original OSCAR trial design. By performing virtual executions of the designs using actual data from the OSCAR trial, we demonstrated how decisions would be made using the posterior predictive probability of trial success at each interim analysis.

OSCAR is a trial where a Bayesian sequential design could have been used as it has a relatively simple design, short follow-up period for the primary outcome, objective primary outcomes that can easily be collected, and slow recruitment. Trials that have longer follow-up periods for the primary outcome and faster recruitment rates are more challenging for adaptive designs as less information may be available at each interim analysis.

Due to the two-armed nature of the study, the design adaptations were restricted to stopping early for success or futility. The proposed Bayesian sequential designs differed in the number and timing of their interim analyses. Bayesian adaptive designs can be most advantageous for trials with complex designs, such as multi-arm trials or those with longitudinal modelling due to their ability to deal with multiple complex decisions [16].

The OSCAR study had planned interim analyses but no formal stopping rules. The early stopping rules in the Bayesian sequential designs for OSCAR allowed for specification of more aggressive stopping and allowed stopping for futility. This is particularly important for interventions that appear to be harmful or are more expensive and enables patients to receive beneficial treatments more quickly. The cost per patient in the OSCAR trial was £3402, and significant savings could have been made by stopping the trial early. The OSCAR trial did not allow for early futility stopping and if it had done so, our Bayesian designs may not have shown an increased efficiency.

Not all Bayesian adaptive trials would want to incorporate early stopping for futility, and the decisions to be made at interim analyses are trial-dependent. For instance, trials that plan cost-effectiveness in their primary analysis may not want to stop early for futility, or may wish to incorporate cost-effectiveness into their stopping criteria.

The simulations of the Bayesian designs produced similar operating characteristics. The virtual re-executions produced similar results to the OSCAR trial across the designs for the primary outcome analysis. In practice, one Bayesian design would have been used and the retrospective comparison of multiple designs would not be possible. Also, designs which stop early have been shown to have small biases in the treatment effect estimates (see, for example, [17, 18]) compared to trials that did not stop early. Checks for potential bias should be performed during the simulation stage and bias-correction methods implemented if necessary (e.g., [19]).

### Limitations

The designs proposed in this paper are situation-specific, as all Bayesian adaptive designs are, and cannot be generalised to all phase III trials. Similar principles to those outlined in this study should be used when constructing adaptive designs that are tailored to the trial aims and clinical practicalities in other settings.

Sequential designs are well known to reduce the expected sample size, and for these trials, similar results might have been obtained if frequentist stopping criteria had been employed. Bayesian criteria were employed to demonstrate their usefulness to clinicians, particularly with regards to interpretability.

The adaptive designs presented in this paper are not necessarily optimal – if different clinicians had been consulted, alternative designs would have been selected. An increase in the number of interims would have led to smaller expected sample sizes, but at the cost of increased operational complexity. Also, if different values had been chosen for the stopping criteria, different decisions may have been made at the interim analyses. For instance, if Design 2 instead used less aggressive stopping boundaries for futility, higher power could be obtained, whilst maintaining the ability to stop early for harm. Selection of the stopping boundaries involves a trade-off of the power, type I error and expected sample size. The main benefit of this approach is that efficient and practically relevant designs can be generated using statistical measures that are intuitive to interpret.

The decisions made by the clinical academics when constructing and choosing the designs were based on a mixture of the statistical aspects of the designs as well as their views about the operational factors. These included the timings of interim analyses and concerns about the perceived external validity of very aggressive stopping rules when presented to a clinical audience who are often naïve to these novel designs. These practical issues are important to consider when such designs are proposed. Operationally, interim analyses need to be adequately spaced to allow time for data cleaning, performing the analysis and presentation to DMECs and Trial Steering Committees (TSCs). In setting the boundaries, it was also noted that decisions need to be taken as to whether the analyses should be based at pre-specified times (which are easier to manage operationally) or a pre-specified number of samples (which are harder to manage operationally, but are easier to manage statistically).

There are a number of practical challenges associated with running adaptive designs in RCTs. In these analyses, we assumed there was no delay between the interim analyses and stopping recruitment if the stopping criteria were met. We also assumed that primary outcome data were immediately available following the 30-day follow-up period. In reality, there might be slight delays for both of these processes, particularly for the former as the DMEC and TSC are likely to be involved. This would decrease the savings achieved by the Bayesian sequential designs.

There are also issues regarding blinding of the TSC and DMEC to interim analysis results, and decisions regarding these must be made before the trial begins, as well as decisions about whether the DMEC and TSC will be bound by the results of the adaptive design. It may be that the decision to stop or continue the trial is made by the DMEC, who then convey the decision to the TSC. The interim analyses themselves should not take long to perform as this can be set-up in advance of the trial and can be automated.

Our Bayesian adaptive designs assumed that stopping early for success or futility was driven by the primary outcome. For OSCAR, the primary outcome was also a safety outcome. If a suitable secondary outcome had been available to use in the interim analyses, then different decisions may have been made when considering the trade-off between outcomes.

Part of the reason for the lack of wide scale adoption of adaptive or sequential designs may be that investigators are not incentivised to terminate trials early. Although early termination may have benefits for the funding agency, it has little benefit for the researchers, who may then face problems of loss of research income and retention of staff. Furthermore, there may be criticism of trials that fail to recruit their planned maximum sample size. It is easier to apply adaptive methodologies in multi-armed trials, where closure of arms is less consequential. It is likely that a change in the funding model for publicly funded trials is required to take full advantage of innovation in trial design.

## Conclusions

There is a great need for phase III trials to become more efficient, yet the majority of clinical trials continue to employ traditional methods. Innovation in clinical trial design is of high importance as it can potentially improve the efficiency, quality of knowledge gained, cost and safety of clinical trials. In this work we have illustrated the benefits of using Bayesian sequential trial designs, using a published example from respiratory medicine, and recommend their use in the wider clinical community.

## Additional files

Additional file 1: Prior distributions. (DOCX 14 kb)

Additional file 2: Distribution plots for sample size and trial duration for OSCAR trial simulations (DOCX 139 kb)

Additional file 3: Simulated operating characteristics for Bayesian sequential designs for different control arm rates (DOCX 22 kb)

## Abbreviations

ARDS: Acute Respiratory distress syndrome
DMEC: Data Monitoring and Ethics Committee
FACTS: Fixed and Adaptive Clinical Trial Simulator
HFOV: High frequency oscillatory ventilation
OSCAR: High Frequency OSCillation in Acute Respiratory distress syndrome study
RCT: Randomised controlled trial
TSC: Trial Steering Committee
