# Supplementary material for: Using Bayesian adaptive designs to improve phase III trials: a respiratory care example
Source: BMC Med Res Methodol. 2019 May 14;19:99. doi: 10.1186/s12874-019-0739-3 (PMC6515675; doi:10.1186/s12874-019-0739-3)
Supplement: Supplementary file 1 — Prior distributions. (DOCX 14 kb) [file 12874_2019_739_MOESM1_ESM.docx]

# Additional File 1 - Prior distributions

Since 30-day mortality is a binary outcome, we used the Bernoulli distribution to model the primary outcome:

$$Y_{j}|t \sim Bernoulli(p_{t})$$

where $Y_{j}$ is the primary outcome for patient *j* and $p_{t}$ is the probability distribution for the primary outcome rate for arm *t*. Since this is a Bayesian approach, we need prior distributions for $p_{t}$.

Priors may be classed as uninformative, which can be used when one has little previous information, or informative, which combine historical evidence or expert opinion with the trial data. Initially, the same uninformative prior was used for both arms, $logit p_{t} \sim N(0, 4$), where$logit p_{t}$ is the log-odds of having the primary outcome in arm *t.* This is roughly the same as having the values of $p_{t}$ being equally likely to occur for each value from 0% to 100%.

A prior sensitivity analysis was conducted in which the operating characteristics of each of the designs were studied under a number of different informative priors, which were derived using information from the trial protocol and systematic reviews available at the time OSCAR was originally designed.

In FACTS, the control arm can be modelled using the same prior distribution as the new treatment or separately, and informative priors can be implemented using fixed normal prior distributions or by using a hierarchical prior for the control arm.

A fixed, weakly informative normal prior was assumed for the control arm, using information from previous studies that was available at the time the trial protocols were written. The mean of the previous studies was used as the mean for the normal distribution (converted to the log-odds scale) and a standard deviation of 1 was used for the prior on the log-odds scale. The above-mentioned uninformative prior was initially used for the high frequency oscillatory ventilation (HFOV) arm. Information from previous studies that used HFOV was also available, and so an informative prior for the HFOV arm was constructed using the means from these studies and a standard deviation of 1 and was used in a separate analysis with the weakly informative prior for the control.

A hierarchical prior was also used for the control arm which combined the information from previous studies by modelling the response on the control as coming from a distribution that also contains some historical response rates. FACTS allows the user to enter the sufficient statistics from the previous studies, such as the number of subjects experiencing the primary outcome in the control arm and the number of subjects observed in the control arm, and the prior distributions for the hyper-parameters in the hierarchical model. FACTS used this information to perform a meta-analysis of the historical trials and current trial using random effects models. One can alter the weighting of the historical information and degree of borrowing via the priors for the hyper-parameters. Weakly informative priors were used for the hyper-parameters. The above-mentioned uninformative prior was used for the HFOV arm.

Optimistic priors were investigated in which the mean response for each arm was centred on the target treatment rates from the original sample size calculations, e.g., 45% vs. 36%. Pessimistic priors were also investigated where the same prior was used for both treatment arms, assuming no difference between treatment arms, centred on the control arm rate from the sample size calculation, i.e., 45%. Both the optimistic and pessimistic priors had standard deviations of 1 (on the log-odds scale for the primary outcome rate).
