# Supplementary material for: Using Bayesian adaptive designs to improve phase III trials: a respiratory care example
Source: BMC Med Res Methodol. 2019 May 14;19:99. doi: 10.1186/s12874-019-0739-3 (PMC6515675; doi:10.1186/s12874-019-0739-3)
Supplement: Supplementary file 2 — Distribution plots for sample size and trial duration for OSCAR trial simulations (DOCX 139 kb) [file 12874_2019_739_MOESM2_ESM.docx]

# Additional File 2 - Distribution plots for sample size and trial duration for OSCAR trial simulations
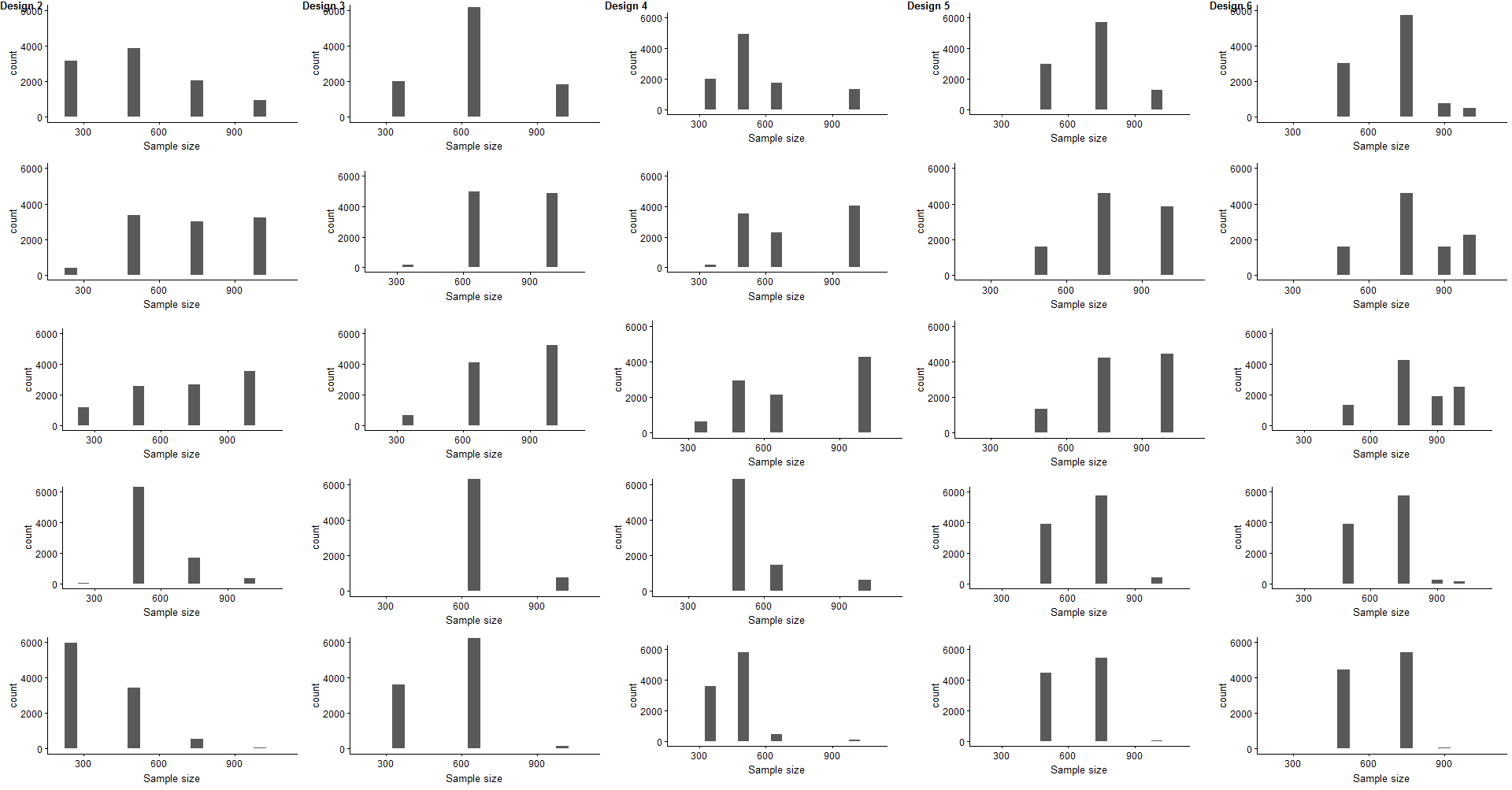


**Figure S1.** Distributions of the sample sizes over the 10, 000 simulations performed for each design (column) and scenario (row) combination. Column 1 is Design 2, Column 2 is Design 3, Column 3 is Design 4, Column 4 is Design 5, and Column 5 is Design 6 from Table 1 in the main paper. Row 1 is “No difference: 45% vs 45%”, Row 2 is “Target difference: 45% vs 36%”, Row 3 is “Small difference: 45% vs 40%”, Row 4 is “Large difference: 45% vs 30%”, and Row 5 is “Treatment harmful: 45% vs 50%”, where the scenarios are given as control arm rate (%) vs HFOV arm rate (%).


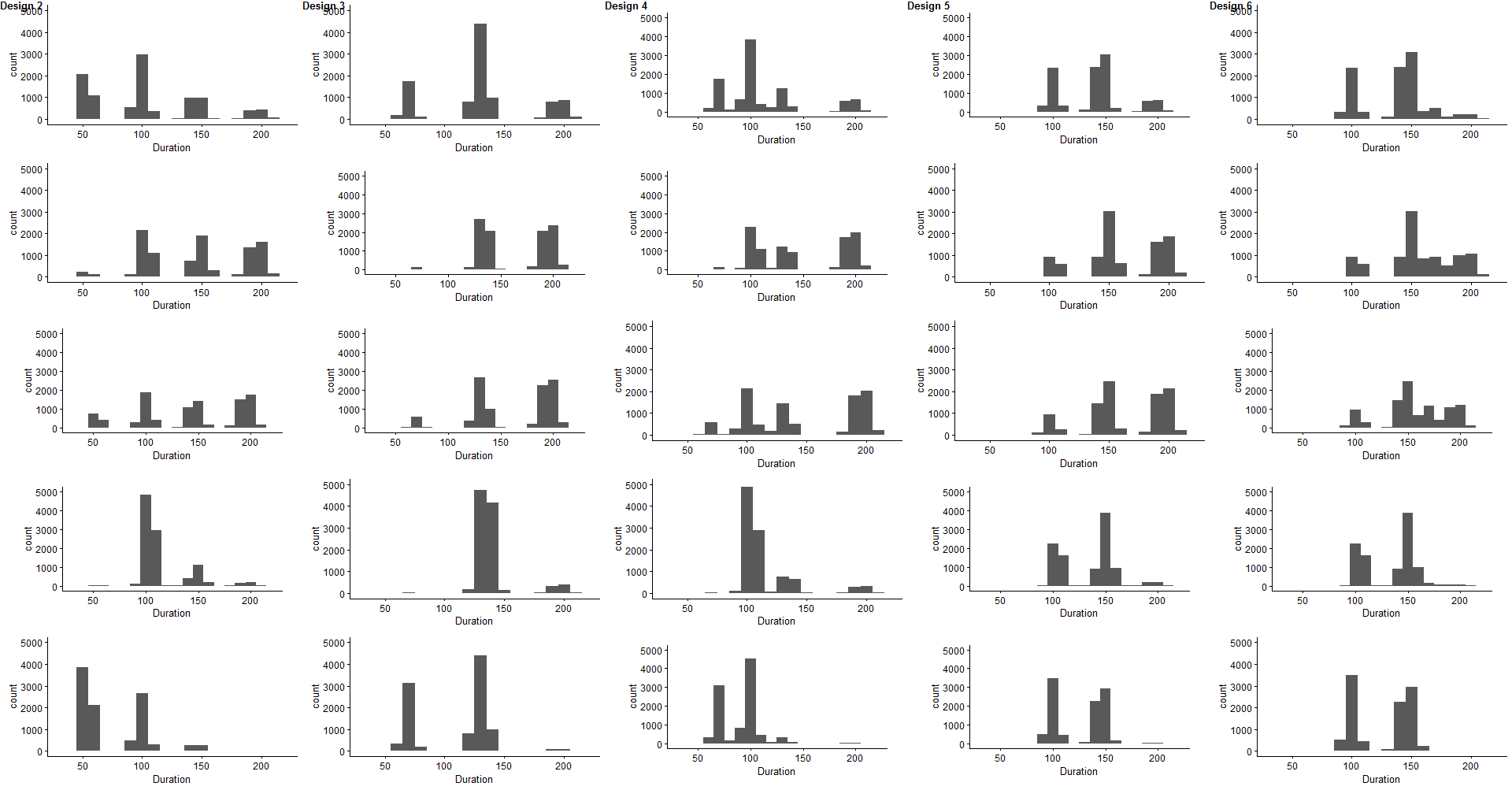


**Figure S2.** Distributions of the trial durations over the 10, 000 simulations performed for each design (column) and scenario (row) combination. Column 1 is Design 2, Column 2 is Design 3, Column 3 is Design 4, Column 4 is Design 5, and Column 5 is Design 6 from Table 1 in the main paper. Row 1 is “No difference: 45% vs 45%”, Row 2 is “Target difference: 45% vs 36%”, Row 3 is “Small difference: 45% vs 40%”, Row 4 is “Large difference: 45% vs 30%”, and Row 5 is “Treatment harmful: 45% vs 50%”, where the scenarios are given as control arm rate (%) vs HFOV arm rate (%)
