# Supplementary material for: Using Bayesian adaptive designs to improve phase III trials: a respiratory care example
Source: BMC Med Res Methodol. 2019 May 14;19:99. doi: 10.1186/s12874-019-0739-3 (PMC6515675; doi:10.1186/s12874-019-0739-3)
Supplement: Supplementary file 3 — Simulated operating characteristics for Bayesian sequential designs for different control arm rates (DOCX 22 kb) [file 12874_2019_739_MOESM3_ESM.docx]

## Additional File 3 – Simulated operating characteristics for Bayesian sequential designs for different control arm rates

**Table S1. Operating characteristics for the proposed Bayesian sequential designs for the OSCAR trial when varying the control arm rate^a^.**

|  | | Average duration (weeks) | Average sample size (SD) | Proportion stopped early for success | Overall Proportion Successful | Proportion stopped early­ for futility |
| --- | --- | --- | --- | --- | --- | --- |
| Design 1: Fixed design | | | | |  |  |
| Target difference: 45% vs 36% | | 196 | 1006 (0) | NA | 0.8219 | NA |
| Smaller control rate: 40% vs 31% | | 196 | 1006 (0) | NA | 0.845 | NA |
| Larger control rate: 50% vs 41% | | 196 | 1006 (0) | NA | 0.8083 | NA |
|  |  |  |  |  |  |  |
| Design 2: Interim analysis at 250, 500 and 750 patients | |  |  |  |  |  |
| Target difference: 45% vs 36% | | 145 | 730 (227) | 0.5319 | 0.7793 | 0.1434 |
| Smaller control rate: 40% vs 31% | | 144 | 727 (224) | 0.5582 | 0.8013 | 0.1257 |
| Larger control rate: 50% vs 41% | | 146 | 735 (225) | 0.5195 | 0.7701 | 0.1496 |
|  |  |  |  |  |  |  |
| Design 3: Interim analysis at 335 and 670 patients | |  |  |  |  |  |
| Target difference: 45% vs 36% | | 163 | 828 (179) | 0.4314 | 0.8079 | 0.0829 |
| Smaller control rate: 40% vs 31% | | 162 | 823 (177) | 0.4564 | 0.8262 | 0.0744 |
| Larger control rate: 50% vs 41% | | 163 | 831 (179) | 0.4177 | 0.7889 | 0.087 |
|  | |  |  |  |  |  |
| Design 4: Interim analysis at 335, 500 and 670 patients | | | | |  |  |
| Target difference: 45% vs 36% | | 147 | 741 (230) | 0.4723 | 0.7873 | 0.1227 |
| Smaller control rate: 40% vs 31% | | 146 | 736 (228) | 0.4967 | 0.8090 | 0.1113 |
| Larger control rate: 50% vs 41% | | 148 | 749 (229) | 0.4593 | 0.7755 | 0.1239 |
|  | |  |  |  |  |  |
|  | |  |  |  |  |  |
| Design 5: Interim analysis at 503 and 755 patients | | | | |  |  |
| Target difference: 45% vs 36% | | 160 | 812 (176) | 0.5237 | 0.813 | 0.0922 |
| Smaller control rate: 40% vs 31% | | 159 | 806 (177) | 0.5492 | 0.8246 | 0.0832 |
| Larger control rate: 50% vs 41% | | 160 | 813 (177) | 0.5157 | 0.7995 | 0.0935 |
|  |  |  |  |  |  |  |
| Design 6: Interim analysis at 503, 755 and 880 patients | |  |  |  |  |  |
| Target difference: 45% vs 36% | | 158 | 792 (159) | 0.6320 | 0.7990 | 0.1415 |
| Smaller control rate: 40% vs 31% | | 156 | 787 (160) | 0.6535 | 0.8153 | 0.1271 |
| Larger control rate: 50% vs 41% | | 155 | 793 (160) | 0.6275 | 0.7912 | 0.1445 |

^a^ For each design results are shown for a range of control arm rates and assuming a target reduction of 9% in the primary outcome rate from the control. This is described in the first column as control vs high-frequency oscillatory ventilation primary outcome rate (as %). The “proportions” in columns 4-6 refer to the proportion of the 10, 000 simulated trials for each scenario, and the averages and standard deviations (SD) are over the 10, 000 simulated trials.
